# Kallikrein-Related Peptidase 12 (KLK12) in Breast Cancer as a Favorable Prognostic Marker

**DOI:** 10.3390/ijms24098419

**Published:** 2023-05-08

**Authors:** Ai Sato, Kiyoshi Takagi, Ayano Yoshimura, Wakana Tsukamoto, Mio Yamaguchi-Tanaka, Yasuhiro Miki, Akiko Ebata, Minoru Miyashita, Takashi Suzuki

**Affiliations:** 1 Department of Pathology and Histotechnology, Tohoku University Graduate School of Medicine, Sendai 980-8575, Miyagi-ken, Japan; ai.sato.b7@tohoku.ac.jp (A.S.); mio.yamaguchi.p3@dc.tohoku.ac.jp (M.Y.-T.); t-suzuki@patholo2.med.tohoku.ac.jp (T.S.); 2 Department of Anatomic Pathology, Tohoku University Graduate School of Medicine, Sendai 980-8575, Miyagi-ken, Japan; miki@patholo2.med.tohoku.ac.jp; 3 Department of Disaster Obstetrics and Gynecology, International Research Institute of Disaster Science, Tohoku University, Sendai 980-8574, Miyagi-ken, Japan; 4 Department of Breast and Endocrine Surgical Oncology, Tohoku University Graduate School of Medicine, Sendai 980-8575, Miyagi-ken, Japan; akiko.ebata@med.tohoku.ac.jp (A.E.); atihsayim8m8@med.tohoku.ac.jp (M.M.); 5 Department of Breast Surgery, Osaki Citizen Hospital, Osaki 989-6183, Miyagi-ken, Japan; 6 Department of Pathology, Tohoku University Hospital, Sendai 980-8574, Miyagi-ken, Japan

**Keywords:** breast cancer, immunohistochemistry, kallikrein-related peptide 12 (KLK12), metastasis, prognosis

## Abstract

Kallikrein-related peptides (KLKs) form an evolutionally conserved subgroup of secreted serine proteases that consists of 15 members (KLK1-15). Previous studies have shown that KLKs regulate diverse biological processes, but the clinical significance of KLKs remains largely unclear in human breast cancers. We examined the expression profile of 15 KLK genes in breast carcinomas using microarray data. Next, we immunolocalized KLK12 in 140 breast carcinomas and evaluated its clinical significance. Subsequently, we examined the effects of KLK12 on proliferation and migration in breast cancer cell lines. From microarray analyses, it turned out that KLK12 was the most strongly associated with low-grade malignancy in breast carcinomas among the 15 KLK members. Immunohistochemical KLK12 status was positively associated with ER and PR status, while it was inversely associated with stage, pathological T factor, lymph node metastasis, and distant metastasis. Prognostic analyses demonstrated that KLK12 was a favorable prognostic factor for both disease-free and breast cancer-specific survival of the patients. Furthermore, the knockdown of KLK12 significantly increased cell proliferation activity and cell migration of breast cancer cells. These results suggest that KLK12 has antitumorigenic effects associated with proliferation and migration and immunohistochemical KLK12 status as a potent favorable prognostic factor in breast carcinoma patients.

## 1. Introduction

Breast cancer is one of the most common malignancies in women, and more than 2 million cases are newly diagnosed around the world [1]. Recent studies revealed that breast cancer is a highly heterogeneous disease, and clinical behavior is quite variable among the patients. Therefore, it is important to explore new biomarkers which precisely predict the clinical outcome of breast cancer.

Kallikrein-related peptides (KLKs) form an evolutionally conserved subgroup of secreted serine proteases that consists of 15 members (i.e., KLK1-15) structurally homologous genes located at the long arm of chromosome 19 [2]. KLK members regulate diverse biological processes and have a crucial involvement in cell proliferation, signaling, migration, and invasion [3]. KLKs are mainly immunolocalized in the nucleus and/or cytoplasm of carcinoma cells [4], and KLKs modulate the progression and metastasis of various carcinomas positively or negatively via a wide range of molecular mechanisms [5]. Emerging evidence suggests the importance of KLKs in breast cancers. For instance, mRNA expression of KLK7 [6], KLK9 [7], KLK13 [8], and KLK15 [9] were reported as favorable markers, while that of KLK5 [10], KLK8 [11] and KLK14 [12] were shown as worse prognostic factors. However, clinical significance has not been comprehensively evaluated among KLK members, although many studies have suggested the possible availability of KLKs as biomarkers in breast cancers. 

Therefore, in this study, we first studied the expression profile of 15 KLK genes in breast carcinomas by microarray data and newly identified that KLK12 was the most strongly associated with low-grade malignancy. KLK12 mRNA expression has been previously examined in breast carcinomas, but the association between KLK12 mRNA and the clinical outcome of breast cancer patients is still controversial [3,13]. Moreover, KLK12 protein has not been examined in breast cancers to the best of our knowledge. Therefore, we subsequently performed immunohistochemistry for KLK12 in breast carcinoma tissues to clarify its clinicopathological significance, and in vitro studies were subsequently performed using breast cancer cells to prove the biological significance of KLK12 in breast cancers.

## 2. Results

### 2.1. Expression Profile of KLK Genes in the Breast Carcinoma

**Figure 1:**
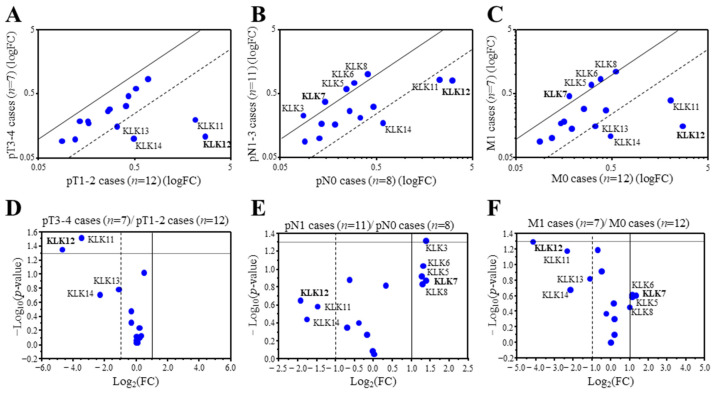
Expression profile of KLK genes in breast carcinoma. (**A**–**C**) Scatter plot analysis of microarray data for 15 KLK genes in 19 breast carcinoma tissues according to the status of pT (**A**), pN (**B**), and M (**C**). KLK genes with a relative expression ratio >2.0 or <0.5 were summarized in each figure. (**D**–**F**) Scatter plot showing the -Log10-*p*-values (y-axis) and the Log2 (fold change) of the 15 KLK genes (pT1,2 cases vs. pT3,4 cases (**D**), pN0 cases vs. pN1 cases (**E**), and M0 cases vs. M1 cases (**F**)). The data points above the lines represent genes having a *p* value < 0.05 and FC ± 1. The gene that showed the highest or lowest ratio was described in bold.

We first examined associations between the expression of KLK genes and the TNM status of 19 breast carcinoma using microarray data. We detected 20 probes corresponding to 15 KLK genes from the microarray data. KLK2, 3, and 6 were duplicated, and KLK13 was triplicated. Therefore the probe with the strongest signal was selected for analysis. When the expression ratio of a gene was >2.0 or <0.50, we tentatively determined that the expression was predominantly high or low in this study [14]. Among the 15 KLK genes, KLK12 (25.2-fold), KLK11 (11.1-fold), KLK14 (4.9-fold), and KLK13 (2.1-fold) were predominantly expressed in lower pT (pT1,2) group compared to the higher pT (pT3,4) group (Figure 1A). On the other hand, KLK12 (3.8-fold), KLK14 (3.4-fold), and KLK11 (2.8-fold) were predominantly expressed in the cases negative for lymph node metastasis (pN0), while KLK7 (0.38-fold), KLK3 (0.38-fold), KLK8 (0.41-fold), KLK6 (0.40-fold) and KLK5 (0.41-fold) were predominantly expressed in cases positive for lymph node metastasis (pN1-3) (Figure 1B). As shown in Figure 1C, KLK12 (17.7-fold), KLK11 (5.2-fold), KLK14 (4.5-fold), and KLK13 (2.2-fold) were predominantly expressed in the cases negative for distant metastasis (M0), while KLK7 (0.40-fold), KLK5 (0.45-fold), KLK6 (0.46-fold) and KLK8 (0.50-fold) were predominantly expressed in the cases positive for distant metastasis (M1) in this study. Figure 1D–F is the volcano plots visualizing the statistical significance versus fold change, indicating that KLK12 was the most strongly expressed in the lower pT (pT1,2) group, lymph node metastasis negative group, and distant metastasis negative group.

Clustering analysis revealed that KLK10-14 genes consisted of one cluster in the 19 breast carcinoma (Supplementary Figure S1), and strongly positive associations was detected among these gene expressions (KLK10 and KLK11: *p* < 0.0001, KLK10 and KLK12: *p* = 0.002, KLK10 and KLK13: *p* = 0.0050, KLK10 and KLK14: *p* < 0.0001, KLK11 and KLK12: *p* < 0.0001, KLK11 and KLK13: *p* < 0.0001, KLK11 and KLK14: *p* < 0.0001, KLK12 and KLK13: *p* < 0.0001, KLK12 and KLK14: *p* < 0.0001, and KLK13 and KLK14: *p* = 0.0060).

These data suggested that KLK12 was most strongly associated with low-grade malignancy of breast carcinoma among the KLK family.

### 2.2. KLK12 Immunolocalization in Human Breast Carcinoma

**Figure 2:**
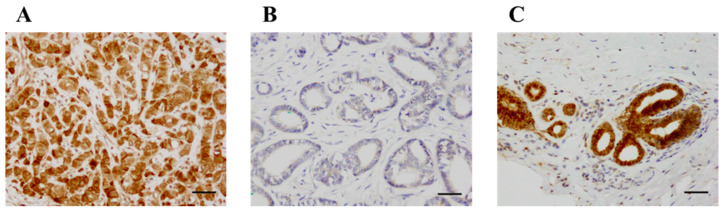
Immunohistochemistry for KLK12 in invasive breast carcinoma. (**A**) KLK12 was immunolocalized in the nucleus and cytoplasm of breast carcinoma cells. (**B**) KLK12-negative case. (**C**) KLK12 immunoreactivity was positive in the epithelium of the mammary gland in normal breast tissue. Bar = 50 μm, respectively.

KLK12 was immunolocalized in the nucleus and cytoplasm of breast carcinoma cells (Figure 2A,B). KLK12 immunoreactivity was positive in the epithelium of non-neoplastic mammary glands but negligible in the stroma (Figure 2C). 

Associations between immunohistochemical KLK12 status and various clinicopathological parameters in breast carcinomas were summarized in Table 1. The number of KLK12-positive cases was 72 out of 140 (51%). The immunohistochemical KLK12 status was positively associated with ER status (*p* = 0.036) and PR status (*p* = 0.0058), while it was inversely associated with the stage (*p* = 0.0007), pathological T factor (pT) (*p* = 0.0002), lymph node metastasis (*p* = 0.030) and distant metastasis (*p* = 0.035) 

### 2.3. Association between KLK12 and Clinical Outcome of Breast Cancer Patients

**Figure 3:**
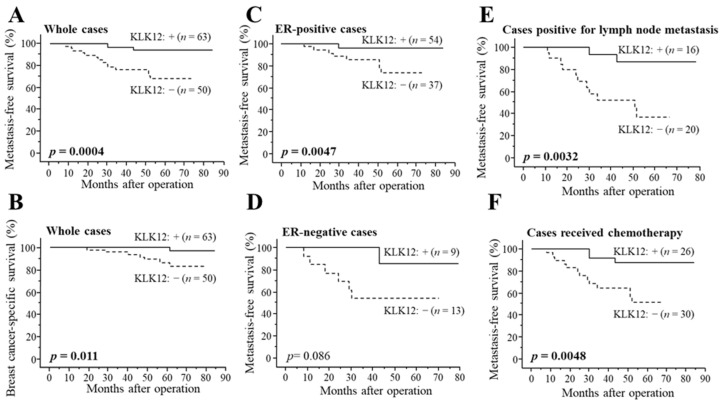
Distant disease-free (**A**,**C**–**F**) and breast cancer-specific survival (**B**) of stage I-III breast cancer patients according to KLK12 status. (**A**,**B**) KLK12 status in whole cases (*n* = 113), (**C**) ER-positive cases (*n* = 91), (**D**) ER-negative cases (*n* = 22), (**E**) cases positive for lymph node metastasis (*n* = 36) and (**F**) cases received chemotherapy (*n* = 56). The solid line shows the KLK12-positive group, and the dashed line shows the KLK12-negative group. *p* values < 0.05 were considered significant and shown in bold.

As demonstrated in Figure 3A, KLK12 status was significantly associated with a decreased incidence of distant metastasis in stage I–III patients (*n* = 113) (*p* = 0.0004). The association between KLK12 status and breast cancer-specific survival was summarized in Figure 3B, and a significant association was detected between KLK12 status and a favorable clinical outcome of patients (*p* = 0.011). Similar tendencies were detected in both ER-positive cases (*p* = 0.0047 for metastasis-free survival (Figure 3C) and *p* = 0.16 for breast cancer-specific survival) and ER-negative cases (*p* = 0.086 for metastasis-free survival (Figure 3D) and *p* value was not evaluated for breast cancer-specific survival because no patient died in KLK12-positive group). A significant association between KLK12 status and the better prognosis was also observed in the cases positive for lymph node metastasis (*p* = 0.0032 for metastasis-free survival (Figure 3E) and *p* = 0.040 for breast cancer-specific survival) or cases that received chemotherapy (*p* = 0.0048 for metastasis-free survival (Figure 3F) and *p* = 0.028 for breast cancer-specific survival).

According to the results of univariate analysis of distant disease-free survival using Cox (Table 2), pT, lymph node metastasis, Ki-67 status, KLK12 status, histological grade, and ER status were significant prognostic factors. Following multivariate analysis revealed that KLK12 status (*p* = 0.0038) turned out to be an independent favorable prognostic factor for metastasis-free survival, besides Ki-67 status (*p* = 0.058) and pT (*p* = 0.097) were marginally significant. As shown in Table 3, univariate analysis for breast cancer-specific survival revealed lymph node metastasis, histological grade, pT, KLK12 status, and ER status as significant prognostic variables, in addition to Ki-67 status as a marginally significant variable. Subsequent multivariate analysis demonstrated that only KLK12 status was an independent favorable prognostic marker (*p* = 0.043). In addition, univariate analysis of breast cancer-specific survival in all cases, including stage IV patients (*n* = 140), showed lymph node metastasis (*p* = 0.0006), pT (*p* = 0.0012), KLK12 status (*p* = 0.0049) and Histological grade (*p* = 0.041) were significant prognostic factors and following multivariate analysis revealed that lymph node metastasis (*p* = 0.032) and KLK12 status (*p* = 0.016) were independent favorable prognostic factors (Supplementary Table S1).

We also verified the association between KLK12 mRNA expression and the survival of breast cancer patients using the Kaplan–Meier plotter (https://kmplot.com/analysis/ (accessed on 21 April 2023)), and KLK12 was suggested as a favorable prognostic factor (Supplementary Figure S2).

### 2.4. Effects of KLK12 on Proliferation and Migration of Breast Carcinoma Cells

**Figure 4:**
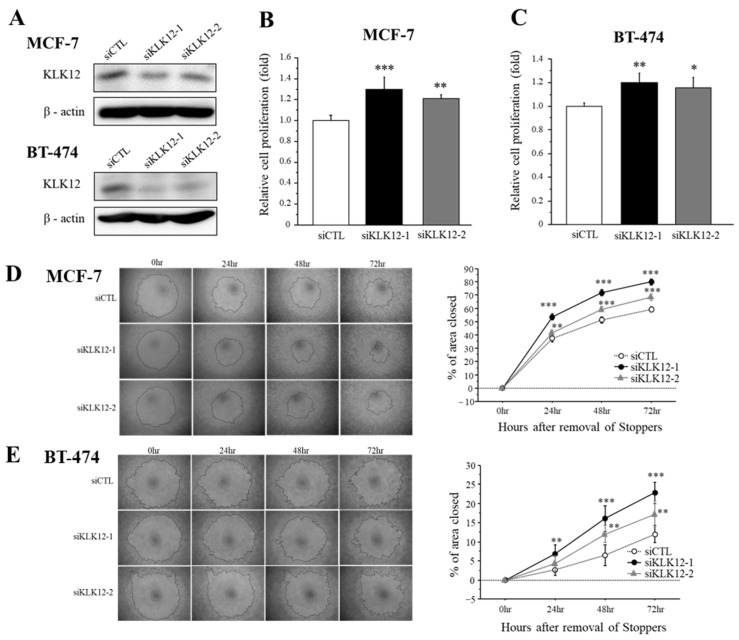
Effects of KLK12 on proliferation and migration in breast carcinoma cells. (**A**) The expression level of KLK12 protein in MCF-7 and BT-474 cells transfected with KLK12-specific siRNA or siCTL. (**B**,**C**) Relative cell proliferation activity of MCF-7 cells (**B**) and BT-474 cells (**C**) transfected with siKLK12-1 and siKLK12-2 (10 nM). Data were summarized as the ratio compared to those transfected with siCTL (3 days after transfection). (**D**,**E**) Wound healing assays in MCF-7 cells (**D**) and BT-474 cells (**E**) transfected with siKLK12-1 and siKLK12-2 (10 nM). Representative photos of the wound healing assay are shown. Relative migration area was evaluated as the ratio (%) to those at the removal of Stoppers (0 h). In all figures, data were presented as mean ± SE (*n* = 3). *; *p* < 0.05, **; *p* < 0.01 and ***; *p* < 0.001.

We transfected specific siRNA for KLK12 in MCF-7 and BT-474 breast cancer cells and performed the cell proliferation assay and migration assay to examine the effects of KLK12 on breast cancer progression. At first, we confirmed that these siRNAs successfully decreased the KLK12 protein level in MCF-7 and BT-474 cells (Figure 4A).

The effects of KLK12 on proliferation activity in breast cancer cells were summarized in Figure 4B (MCF-7) and Figure 4C (BT-474). Cell proliferation was significantly increased in MCF-7 cells transfected with siKLK12-1 and siKLK12-2, compared to those transfected with siCTL, 3 days after the transfection. A similar tendency was detected in BT-474 cells under the same condition.

Cell migration was examined by wound healing assay using MCF-7 cells (Figure 4D) and BT-474 cells (Figure 4E). Relative cell migration was significantly increased in both MCF-7 and BT-474 cells.

We also assessed the WST and wound healing assay using MDA-MB-231 cells which shows a low endogenous expression of KLK12 (Figure S3A). As shown in Figure S3B, the KLK12 protein was markedly increased by the transfection of the KLK12 expressing vector, and KLK12 expressing plasmid overexpression showed a significant decrease in proliferation and migration compared to the control plasmid (Figure S3C,D).

## 3. Discussion

This is the first study to demonstrate the gene expression profile of the KLK family in breast carcinoma tissues. In this study, KLK11-14 genes were predominantly expressed in low-grade malignancies of breast carcinomas. Previous studies demonstrated that KLKs are involved in cancer progression positively or negatively via a wide range of molecular mechanisms [15]. Among the KLK11-14, KLK13 expression was reported as a favorable prognostic marker in breast cancers [8] and ovarian cancers [16] which is consistent with our present results. KLK11 expression was inversely associated with tumor grade in breast carcinomas [17], although the association between KLK11 and prognosis has not been reported in breast cancers. On the contrary, KLK14 mRNA expression was reported as a poor prognostic marker in breast cancers [12]. KLK12 expression was the most pronouncedly suppressed in the breast carcinomas with high-grade malignancy in this study.

Our present microarray analysis also revealed that the expression of KLK10-14 genes consisted of one cluster. KLK4-14 genes sequentially and closely reside at chromosomal region 19q13.41, while KLK1-3 and KLK15 genes are located at 19q13.33. KLK can activate another KLK, and Memari et al. [18] reported that KLK12 activated KLK11 zymogen. Additionally, Gong et al. [13] revealed that the mRNA expression level of KLK12 was positively correlated with that of KLK10 and KLK11 in triple-negative breast cancer. Therefore, KLK12 may participate in the enzymatic cascade in breast carcinoma in cooperation with other KLK members.

This is the first study that immunolocalized KLK12 in breast carcinomas. In this study, KLK12 immunoreactivity was detected in 51% of breast carcinomas, while it was positive in the epithelium of morphologically normal mammary glands. Previously, Yousef et al. [19] reported that KLK12 mRNA level was downregulated in the breast carcinoma tissues compared to the normal breast tissues. Followingly, they indicated that expression of KLK5, 6, 8, and 12 genes was downregulated in the breast carcinoma tissues compared to normal breast tissues [20], which suggests imbalanced expression of KLK members is associated with the development of breast cancers. Our present results seem to be consistent with the previous findings.

In this study, KLK12 immunoreactivity was significantly associated with ER and PR status in breast carcinomas. Previously, Yousef et al. [19] reported that KLK12 mRNA expression was increased by sex hormones (i.e., estrogen, progesterone, and androgen) in BT-474 and T-47D breast cancer cells, which agrees with our present results. Additionally, KLK11 was identified as one of the estrogen-induced genes in MCF-7 breast cancer cells [21]. Since estrogen-mediated transactivation varies among the target genes [22], highly malignant breast carcinomas may more efficiently induce the genes promoting aggressiveness by estrogen rather than KLK12.

Our present study demonstrated that KLK12 status was an independent favorable prognostic factor for both metastasis-free survival and breast cancer-specific survival of breast cancer patients. Previously, Papachristopoulou et al. [3] reported that mRNA expression of KLK12 splice variants (KLK12sv1/2 and KLK12sv3) were good prognosis markers in breast carcinomas, which agrees with our present results because the KLK12 antibody and KLK12 specific siRNA used in this study recognizes these variants. Recently, Gong et al. [13] showed that KLK12 mRNA expression was remarkably associated with shortened survival in triple-negative breast cancer patients. Although KLK12 immunoreactivity tended to be associated with better prognosis also in the triple-negative type (*n* = 15; *p* = 0.13 for metastasis-free survival, and *p* value was not estimated because no patient died in the KLK12-positive group) in this study, a larger sample set is needed to clarify the significance of KLK12 protein in the triple-negative type. The biological function of KLK12 has been unknown in breast cancers. However, KLK5 has been reported as a suppressor of breast cancers, inhibiting the mevalonate pathway [23] and inducing miRNA-mediated antioncogenic pathways [24]. Our present in vitro experiments demonstrated that KLK12 knockdown increased cell proliferation and migration in MCF-7 and BT-474 cells, while overexpression of KLK12 decreased it in MDA-MB-231 cells. KLK12 was therefore considered to play a suppressive role in breast carcinomas. However, it is also true that the effect of KLK12 knockdown on cell proliferation is slight, and the tumor-suppressive role of KLK12 has not been confirmed by in vivo experiments in the present study, while previous in vivo experiments demonstrated decreased colorectal tumor volume by knockdown of KLK12 [25]. The role of KKL12 seems different among the tissues, and further examination will be warranted for a better understanding of the biological significance of KLK12 in breast cancer.

In summary, we examined the gene expression profile of the KLK family in breast carcinomas by microarray analysis and newly identified that KLK12 was the most strongly associated with low-grade malignancy. Subsequent immunohistochemical analysis demonstrated that KLK12 immunoreactivity was positive in the epithelium of non-neoplastic mammary glands, and it was positive in 51% of breast carcinomas. The KLK12 status was positively associated with ER and PR status, while it was inversely associated with stage, pT, lymph node metastasis, and distant metastasis. Moreover, the multivariate analysis turned out that the KLK12 status was an independent favorable prognostic factor for both metastasis-free and breast cancer-specific survival of the patients. Following in vitro, experiments revealed that KLK12 knockdown increased cell proliferation and migration in breast cancer cells. These findings suggest that KLK12 plays an important suppressive role in breast cancers, and immunohistochemical KLK12 status is a potent favorable prognostic factor.

## 4. Materials and Methods

### 4.1. Microarray Analysis

Microarray data of estrogen receptor (ER)-positive breast carcinomas (*n* = 19) were used in the present study, which had been assembled in our previous study [14] (Mayama et al. 2018). Total RNA was extracted from 19 snap-frozen specimens using an RNeasy Mini Kit (QIAGEN, Hilden, Germany). cy3-labeled cRNA synthesis was performed according to the manufacturer’s protocol and hybridized on SurePrint G3 Human GE 8 × 60 K v2 (G4851A, ID 028004 (Agilent Technologies, Waldbronn, Germany)).

### 4.2. Patients and Tissues

One hundred forty specimens of invasive ductal carcinoma, not otherwise specified, of the breast were obtained from Japanese female patients (age range; 27–87 years) who had undergone surgical treatment. All the specimens had been fixed in 10% formalin and embedded in paraffin wax. Among them, stage IV cases (*n* = 27) were obtained from 2000 to 2015 from Tohoku University Hospital (Sendai, Japan) and Osaki Citizen Hospital (Osaki, Japan). Stage I–III patients (*n* = 113) were successively treated at Tohoku University Hospital from 2007 to 2008. Among these 113 patients, 56 patients had received adjuvant chemotherapy, and 91 patients had received adjuvant endocrine therapy. The clinical outcome was evaluated by metastasis-free survival, which was defined as the time from primary surgery until the first event of distant metastasis, and breast cancer-specific survival of stage I-III patients. The mean follow-up time was 61 months (3–91 months). The research protocol was approved by the Ethics Committee at the Tohoku University School of Medicine and the review board of Osaki Citizen Hospital.

### 4.3. Immunohistochemistry

We purchased a monoclonal antibody for KLK12 (clone 364932) from R&D Systems (Minneapolis, MN, USA) and a mouse monoclonal antibody for Ki-67 (MIB1) from DAKO (Carpinteria, CA, USA). We used a Histofine Kit (Nichirei Biosciences, Tokyo, Japan), employing the streptavidin-biotin amplification method, for immunohistochemistry. The antigen-antibody complex was visualized with a 3,3′-diaminobenzidine (DAB) solution with hematoxylin. We used human pancreatic tissue as a positive control based on data of KLK12 in The Human Protein Atlas (https://www.proteinatlas.org/ENSG00000186474-KLK12 (accessed on 12 October 2017)).

Immunohistochemistry for ER (CONFIRM anti-ER (SP1)) and progesterone receptor (PR: CONFIRM anti-PR (1E2); Roche Diagnostics Japan, Tokyo, Japan) was performed by Ventana Benchmark XT (Roche Diagnostics Japan), and that for HER2 was performed using HercepTest (DAKO).

### 4.4. Scoring of Immunohistochemistry

KLK12 was immunolocalized in the nucleus and cytoplasm of carcinoma cells, and the cases that had more than 10% positive carcinoma cells were considered positive [26]. ER, PR and Ki-67 were immunolocalized in the nucleus, and the percentage of immunoreactivity (labeling index; LI) was determined. Cases with ER or PR LI of more than 1% were considered ER-positive or PR-positive breast carcinoma, according to a previous report [27]. HER2 immunostaining was scored according to the standardized HercepTest scoring system (score 0–3) (DAKO), and a score of 3 was considered positive. HER2 gene amplification was also investigated by fluorescence in situ hybridization (FISH) in the score 2 cases, and the cases that showed positive for FISH were also considered positive for HER2. Ki-67 LI was classified into two groups in the uni-and multi-variate analyses using 20% as a cut-off value [28,29].

An intrinsic subtype of breast carcinoma was defined according to the 2011 St Gallen surrogate definition [30] as follows: luminal A (ER and/or PR positive, HER2 negative, Ki-67 LI < 14%), luminal B (ER and/or PR positive, HER2 negative, Ki-67 LI ≥ 14% (HER2 negative), or ER and/or PR positive, HER2 positive (HER2 positive)), HER2 positive (ER and PR negative, HER2 positive), and triple-negative (ER, PR, HER2 negative).

### 4.5. Cell Lines

Human breast cancer cell lines MCF-7, BT-474, and MDA-MB-231 were obtained from the Japanese Collection of Research Bioresources Cell Bank (Osaka, Japan) and American Type Culture Collection (ATCC; Manassas, VA, USA), respectively. These cells were cultured in RPMI-1640 (Fujifilm Wako Chemicals, Osaka, Japan) containing 10% fetal bovine serum (FBS; Biosera, Boussens France).

### 4.6. Small Interfering RNA (siRNA) Transfection

Two siRNA oligonucleotides for KLK12 were used in this study, which was designed as follows: siKLK12-1 (5′-AAACAGUGACAGCCACGUATT-3′) and siKLK12-2 (5′-GCCCUUCUAAGACCCACGATT-3′). MISSION siRNA Universal Negative Control (Sigma-Aldrich, St. Louis, MO, USA) was used as a negative control (siCTL). The siRNAs were transfected using Lipofectamine RNAiMAX Transfection Reagent (Thermo Fisher Scientific, Waltham, MA, USA).

### 4.7. Plasmid Transfection

The FLAG-tagged KLK12 expression plasmid (pKLK12-FLAG) was purchased from GenScript Japan (Tokyo, Japan). As a control, a FLAG-tagged GFP (GFP-FLAG) expression vector was used in this study. The plasmid was transfected into MDA-MB-231 cells using Avalanche-Everyday Transfection Reagent (APRO Science, Tokushima, Japan).

### 4.8. RT-PCR

Total RNA was extracted using TRI reagent (Molecular Research Center, Cincinnati, OH, USA), and cDNA was synthesized using a Rever Tra Ace qPCR RT Master Mix with gDNA Remover (Toyobo, Osaka, Japan). PCR was carried out using the THUNDERBIRD SYBR qPCR Mix (Toyobo). The primer sequences of KLK12 and RPL13A were: KLK12, 5′-GCCTCAACCTCTCCATCGTC-3′ (forward) and 5′-CTTGAAGGACTCCCCCACAC-3′ (reverse); and RPL13A, 5′-CCTGGAGGAGAAGAGGAAAGAGA-3′ (forward) and 5′-TTGAGGACCTCTGTGTATTTGTCAA-3′ (reverse).

### 4.9. Immunoblotting

Total protein was extracted by M-PER Mammalian Protein Extraction Reagent (Pierce Biotechnology, Rockford, IL, USA) with Protease Inhibitor Cocktail (Sigma-Aldrich, St. Louis, MO, USA). The lysate proteins (5 μg) were subjected to SDS-PAGE (10% acrylamide gel) and transferred onto Hybond PVDF membranes (GE Healthcare, Buckinghamshire, UK). The primary anti-KLK12 antibody used was the same as that in the immunohistochemistry, and the anti-FLAG antibody was obtained from FUJIFILM Wako (Osaka, Japan). Anti-b-actin antibody (A3854, Sigma-Aldrich) was used as an internal control. Antibody-protein complexes on the membrane were detected using ImmunoStar LD (Fujifilm Wako Chemicals) and visualized by a LAS-4000 image analyzer (Fuji Photo Film Co., Tokyo, Japan).

### 4.10. Cell Proliferation and Wound Healing Assay

The cell proliferation status of MCF-7, BT-474, and MDA-MB-231 cells was measured by a Cell Counting Kit-8 (Dojindo Molecular Technologies, Kumamoto, Japan). The cell migration property of MCF-7, BT-474, and MDA-MB-231 cells was evaluated by wound healing assay using an Oris cell migration assay kit (Platypus Technologies, Madison, WI, USA), and the remaining gaps were quantified using ImageJ 1.53k software (NIH, Bethesda, MD, USA).

### 4.11. Statistical Analysis

THE ASSOCIATION between the immunohistochemical status of KLK12 and clinicopathological factors was evaluated using the Student’s t-test or a cross-table using the χ^2^-test. Metastasis-free and breast cancer-specific survival curves were generated by the Kaplan-Meier method, and statistical significance was calculated using the log-rank test. Univariate and multivariate analyses were evaluated using a proportional hazard model (Cox).

For comparisons between the two groups, Welch’s *t*-tests were used. *p* value < 0.05 and 0.05 ≤ *p* value < 0.10 were considered significant and borderline significant in this study. The statistical analyses were performed using the StatView 5.0J software (SAS Institute, Cary, NC, USA) in this study.

## Figures and Tables

**Table 1:** Association between immunohistochemical KLK12 status and clinicopathological factors in 140 breast carcinomas.

	KLK12 Status		*p* Value
	Positive (*n* = 72)	Negative (*n* = 68)	
Age † (years)	56.1 ± 1.4	56.6 ± 1.6	0.84
Menopausal status			
Premenopausal	27	24	
Postmenopausal	45	44	0.79
Stage			
I	44	22	
II–IV	28	46	**0.0007**
Pathological T factor (pT)			
pT1	52	28	
pT2-4	20	40	**0.0002**
Lymph node metastasis			
Positive	24	35	
Negative	48	33	**0.030**
Distant metastasis			
Positive	9	18	
Negative	63	50	**0.035**
Histological grade			
1–2	55	54	
3	17	14	0.67
ER status			
Positive	63	50	
Negative	9	18	**0.036**
PR status			
Positive	56	38	
Negative	16	30	**0.0058**
HER2 status			
Positive	10	13	
Negative	62	55	0.40
Ki-67 LI † (%)	14.7 ± 1.7	18.2 ± 2.3	0.21
Intrinsic subtype			
Luminal A	43	30	
Luminal B	20	22	
HER2 positive	3	7	
Triple negative	6	9	0.21

†; Data are presented as mean ± SEM. All other values represent the number of cases. *p* value < 0.05 was significant (in bold).

**Table 2:** Univariate and multivariate analyses of metastasis-free survival in 113 stage I–III breast cancer patients.

	Univariate	Multivariate	
Variable	*p* Value	*p* Value	Relative Risk (95% CI)
pT			
(pT2-4/pT1)	**0.0002** †	*0.097*	3.87 (0.78–19.15)
Lymph node metastasis			
(positive/negative)	**0.0003** †	0.30	2.24 (0.49–10.20)
Ki-67 status			
(≥20%/<20%)	**0.0015** †	*0.058*	3.35 (0.96–11.63)
KLK12 status			
(positive / negative)	**0.0024** †	**0.0038**	0.13 (0.03–0.52)
Histological grade			
(3/1-2)	**0.0085** †	0.54	1.49 (0.42–5.31)
ER status			
(positive/negative)	**0.019** †	0.95	1.04 (0.30–3.66)
HER2 status			
(positive/negative)	0.63		

Statistical analysis was evaluated by a proportional hazard model (Cox). *p* value < 0.05 and 0.05 ≤ *p* value < 0.10 were considered significant and borderline significant and were listed in bold and italics, respectively. †; Significant (*p* < 0.05) and borderline-significant (0.05 ≤ *p* < 0.10) values were examined in the multivariate analyses in this study. 95% CI, 95% confidence interval.

**Table 3:** Univariate and multivariate analyses of breast cancer-specific survival in 113 stage I–III breast cancer patients.

	Univariate	Multivariate	
Variable	*p* Value	*p* Value	Relative Risk (95% CI)
Lymph node metastasis			
(positive/negative)	**0.011** †	0.13	7.42 (0.55–101.06)
Histological grade			
(3/1-2)	**0.018** †	0.32	2.32 (0.43–12.44)
pT			
(pT2-4/pT1)	**0.020** †	0.85	1.22 (0.16–9.54)
KLK12 status			
(positive/negative)	**0.038** †	**0.043**	0.09 (0.01–0.93)
ER status			
(positive/negative)	**0.046** †	0.73	0.73 (0.12–4.38)
Ki-67 status			
(≧20%/<20%)	*0.060* †	0.47	2.10 (0.28–15.48)
HER2 status			
(positive/negative)	0.76		

Statistical analysis was evaluated by a proportional hazard model (Cox). *p* value < 0.05 and 0.05 ≤ *p* value < 0.10 were considered significant and borderline significant and were listed in bold and italics, respectively. †; Significant (*p* < 0.05) and borderline-significant (0.05 ≤ *p* < 0.10) values were examined in the multivariate analyses in this study. 95% CI, 95% confidence interval.

## Data Availability

All data and materials presented in this article and in the Supplementary Materials are available from the corresponding author at reasonable request.

## Supplementary Materials

The supporting information can be downloaded at: https://www.mdpi.com/article/10.3390/ijms24098419/s1.

## Abbreviations

KLK	kallikrein-related peptide
LI	labeling index
PR	progesterone receptor
pT	pathological T factor
