# Supplementary material for: Kallikrein-Related Peptidase 12 (KLK12) in Breast Cancer as a Favorable Prognostic Marker
Source: Int J Mol Sci. 2023 May 8;24(9):8419. doi: 10.3390/ijms24098419 (PMC10179240; doi:10.3390/ijms24098419)
Supplement: Supplementary file 1 [file ijms-24-08419-s001.zip › KLK12 Fig S1.pptx]

## Slide 1
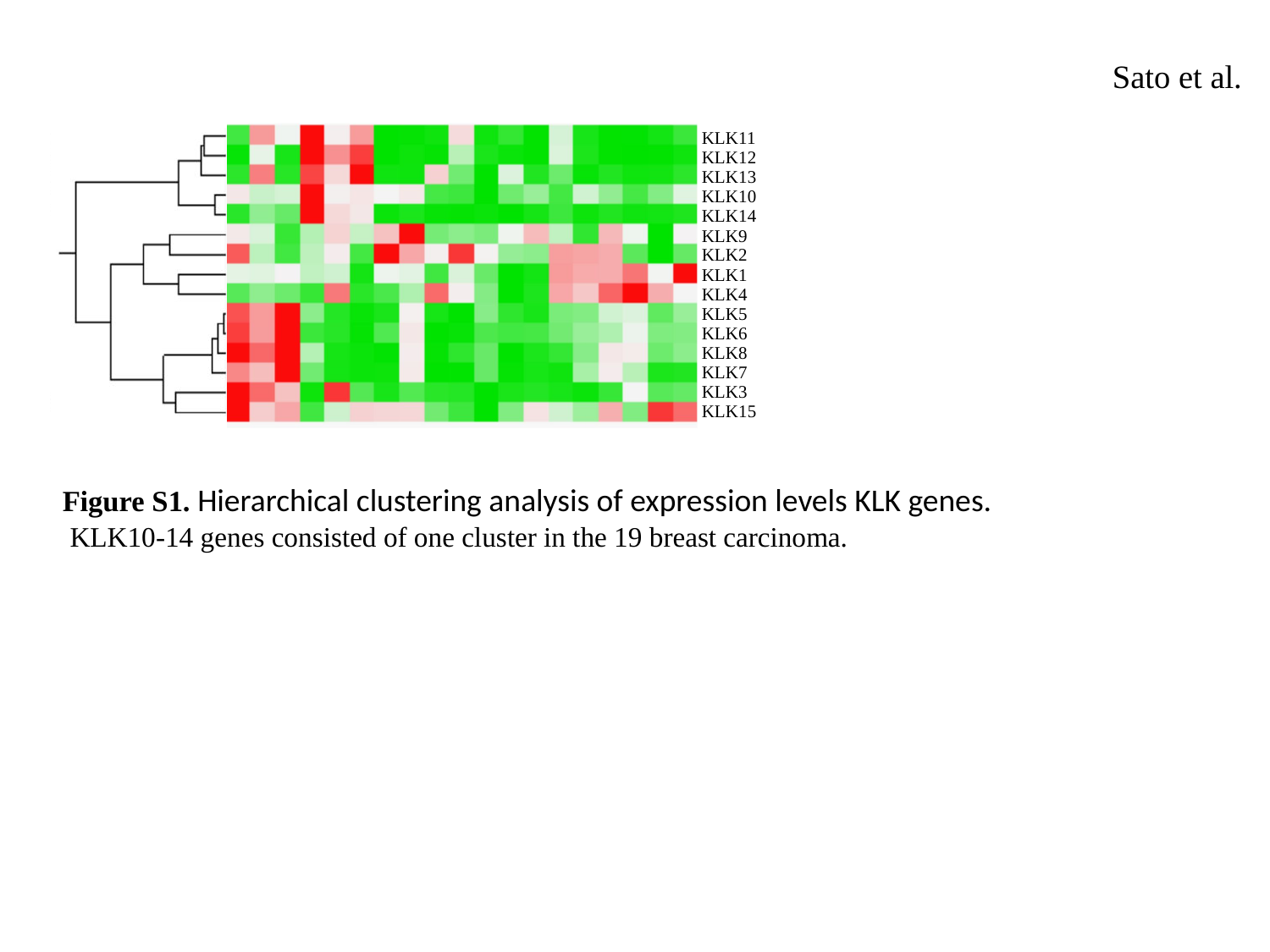

Sato et al.
KLK11
KLK12
KLK13
KLK10
KLK14
KLK9
KLK2
KLK1
KLK4
KLK5
KLK6
KLK8
KLK7
KLK3
KLK15
Figure S1. Hierarchical clustering analysis of expression levels KLK genes.
 KLK10-14 genes consisted of one cluster in the 19 breast carcinoma.
