# Supplementary material for: Kallikrein-Related Peptidase 12 (KLK12) in Breast Cancer as a Favorable Prognostic Marker
Source: Int J Mol Sci. 2023 May 8;24(9):8419. doi: 10.3390/ijms24098419 (PMC10179240; doi:10.3390/ijms24098419)
Supplement: Supplementary file 1 [file ijms-24-08419-s001.zip › KLK12 Fig S2.pptx]

## Slide 1
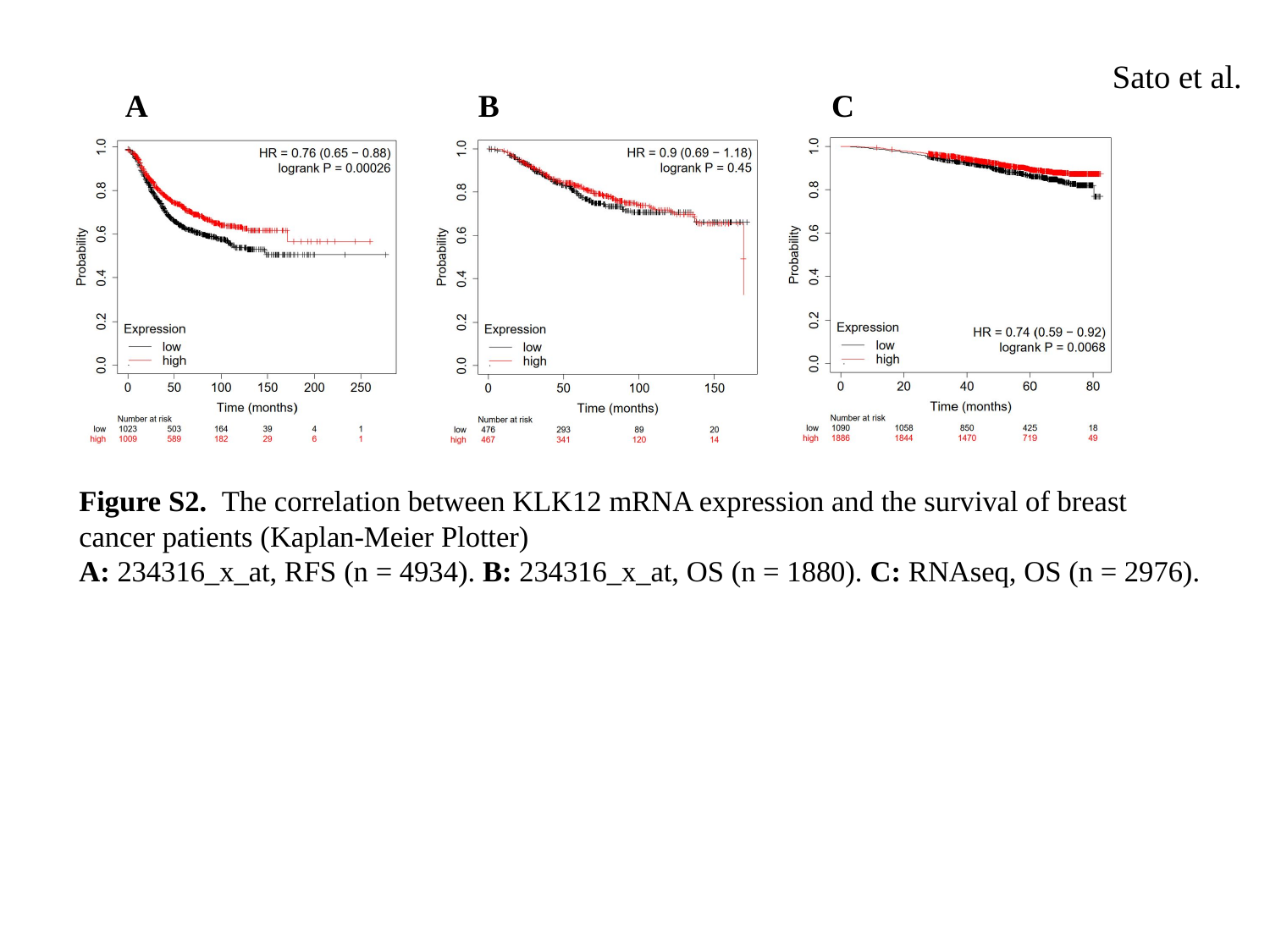

Sato et al.
A
B
C
Figure S2. The correlation between KLK12 mRNA expression and the survival of breast cancer patients (Kaplan-Meier Plotter)
A: 234316_x_at, RFS (n = 4934). B: 234316_x_at, OS (n = 1880). C: RNAseq, OS (n = 2976).
