# Supplementary material for: Kallikrein-Related Peptidase 12 (KLK12) in Breast Cancer as a Favorable Prognostic Marker
Source: Int J Mol Sci. 2023 May 8;24(9):8419. doi: 10.3390/ijms24098419 (PMC10179240; doi:10.3390/ijms24098419)
Supplement: Supplementary file 1 [file ijms-24-08419-s001.zip › KLK12 Fig S3.pptx]

## Slide 1
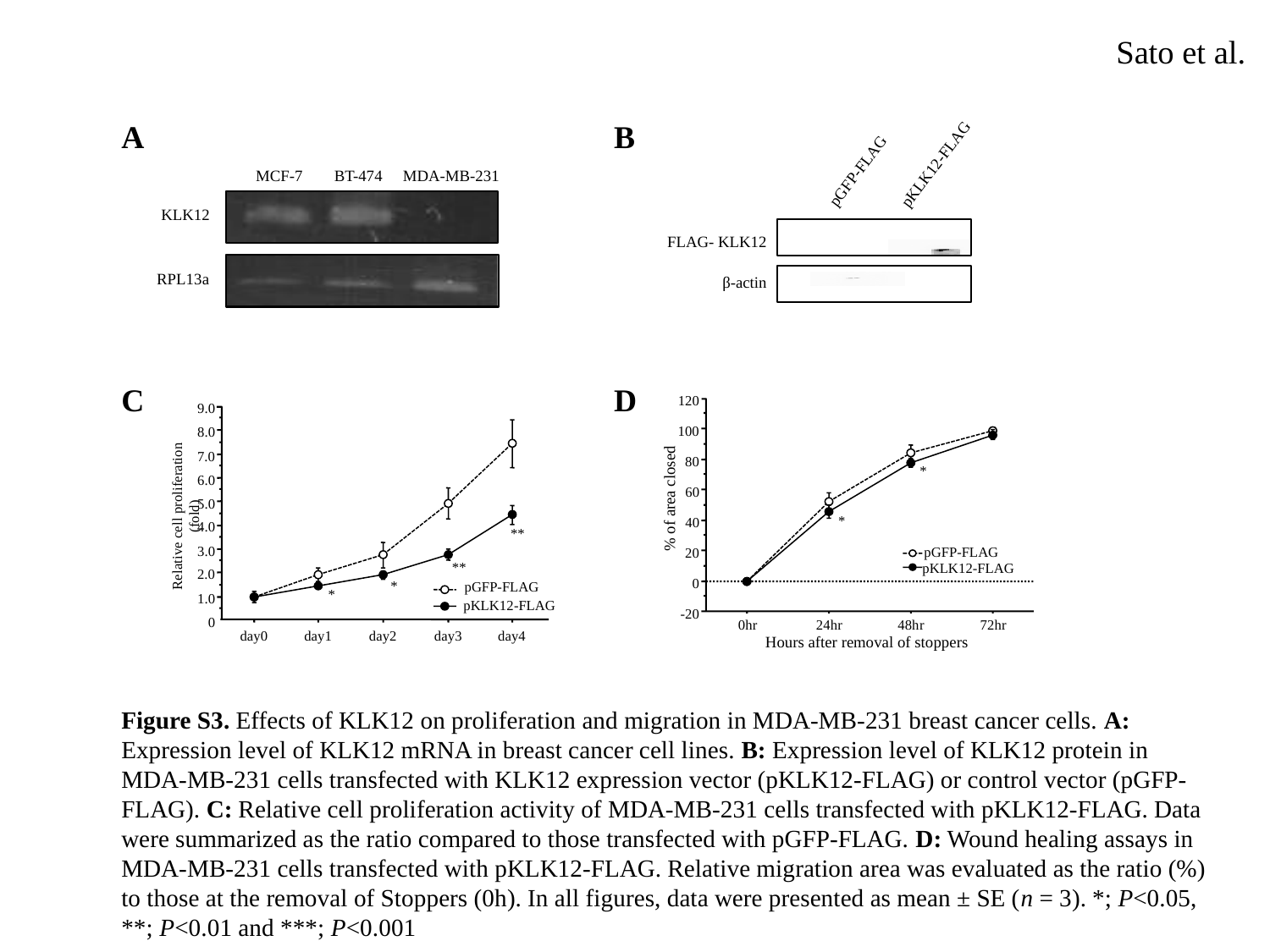

Sato et al.
pKLK12-FLAG
pGFP-FLAG
FLAG- KLK12
β-actin
A
B
MCF-7
BT-474
MDA-MB-231
KLK12
RPL13a
C
D
120
100
80
*
60
% of area closed
*
40
pGFP-FLAG
20
pKLK12-FLAG
0
-20
0hr
24hr
48hr
72hr
Hours after removal of stoppers
9.0
8.0
7.0
6.0
5.0
Relative cell proliferation (fold)
4.0
**
3.0
**
2.0
*
pGFP-FLAG
pKLK12-FLAG
*
1.0
0
day0
day1
day2
day3
day4
Figure S3. Effects of KLK12 on proliferation and migration in MDA-MB-231 breast cancer cells. A: Expression level of KLK12 mRNA in breast cancer cell lines. B: Expression level of KLK12 protein in MDA-MB-231 cells transfected with KLK12 expression vector (pKLK12-FLAG) or control vector (pGFP-FLAG). C: Relative cell proliferation activity of MDA-MB-231 cells transfected with pKLK12-FLAG. Data were summarized as the ratio compared to those transfected with pGFP-FLAG. D: Wound healing assays in MDA-MB-231 cells transfected with pKLK12-FLAG. Relative migration area was evaluated as the ratio (%) to those at the removal of Stoppers (0h). In all figures, data were presented as mean ± SE (n = 3). *; P<0.05, **; P<0.01 and ***; P<0.001
