# Supplementary material for: Kallikrein-Related Peptidase 12 (KLK12) in Breast Cancer as a Favorable Prognostic Marker
Source: Int J Mol Sci. 2023 May 8;24(9):8419. doi: 10.3390/ijms24098419 (PMC10179240; doi:10.3390/ijms24098419)
Supplement: Supplementary file 1 [file ijms-24-08419-s001.zip › KLK12 Table S1.pdf]

Table S1. Univariate and multivariate analyses of breast cancer-specific survival in breast cancer patients (n = 140).

	Univariate	Multivariate	
Variable	*P* value	*P* value	Relative risk (95% CI)
Lymph node metastasis			
(positive/negative)	**0.0006†**	**0.032**	4.92 (1.15-21.06)
pT			
(pT2-4 / pT1)	**0.0012†**	0.36	1.81 (0.51-6.45)
KLK12 status			
(positive/negative)	**0.0049†**	**0.016**	0.21 (0.059-0.75)
Histological grade			
(3 / 1,2)	**0.041†**	0.43	1.49 (0.56-3.96)
ER status			
(positive/negative)	0.15		
Ki-67 status			
(≧20% / <20%)	0.23		
HER2 status			
(positive/negative)	0.93		

Statistical analysis was evaluated by a proportional hazard model (Cox).
P value < 0.05 were considered significant and were listed in bold.
†; Significant (*P* < 0.05) values were examined in the multivariate analyses in this study.
95% CI, 95% confidence interval.
